# External data required timely response by the Trial Steering-Data Monitoring Committee for the NALoxone InVEstigation (N-ALIVE) pilot trial

**DOI:** 10.1016/j.conctc.2017.01.006

**Published:** 2017-01-18

**Authors:** Sheila M. Bird, John Strang, Deborah Ashby, John Podmore, J. Roy Robertson, Sarah Welch, Angela M. Meade, Mahesh K.B. Parmar

**Affiliations:** a MRC Biostatistics Unit, Cambridge CB2 0SR, UK; b National Addiction Centre, King's College London, London SE5 8BB, UK; c Imperial Clinical Trials Unit School of Public Health, London W12 7RH, UK; d Durham University School of Applied Social Sciences, Durham DH1 3LE, UK; e Edinburgh University Usher Institute of Population Health Sciences and Informatics, Edinburgh EH16 4UX, UK; f Turning Point, Gloucester GL1 2HT, UK; g MRC Clinical Trials Unit at University College London, London WC2B 6NH, UK

**Keywords:** Data Monitoring Committee, Randomized pilot trial, External evidence, Elicitation, Causality, Cessation

## Abstract

The prison-based N-ALIVE pilot trial had undertaken to notify the Research Ethics Committee and participants if we had reason to believe that the N-ALIVE pilot trial would not proceed to the main trial. In this paper, we describe how external data for the third year of before/after evaluation from Scotland's National Naloxone Programme, a related public health policy, were anticipated by eliciting prior opinion about the Scottish results in the month prior to their release as official statistics. We summarise how deliberations by the N-ALIVE Trial Steering-Data Monitoring Committee (TS-DMC) on N-ALIVE's own interim data, together with those on naloxone-on-release (NOR) from Scotland, led to the decision to cease randomization in the N-ALIVE pilot trial and recommend to local Principal Investigators that NOR be offered to already-randomized prisoners who had not yet been released.

## 1 Introduction

Naloxone is an opioid antagonist used for emergency resuscitation following opioid overdose. Prisoners with a history of heroin use by injection have a high risk of drug-related death (DRDs) in the first weeks after release from prison [1], [2], [3]. The N-ALIVE trial was planned as a large prison-based randomized controlled trial (RCT) to test the effectiveness of Naloxone-on-release (NOR) in the prevention of fatal opiate overdoses soon after release (30% reduction in the first 4-weeks; 20% in weeks 5–12) [4]. The N-ALIVE pilot trial (ISRCTN34044390) was a randomized feasibility study to test the main trial's assumptions on recruitment of prisons and prisoners, and also the logistics for ensuring that randomized participants received their N-ALIVE pack on release [5]. See Meade et al. [6] for how delivery of the N-ALIVE protocol was achieved in 16 prisons in England. See Parmar et al. [5] for the feasibility outcomes in the N-ALIVE pilot trial. The N-ALIVE pilot trial had undertaken to notify the Research Ethics Committee and participants if we had reason to believe that the N-ALIVE pilot trial would not proceed to the N-ALIVE main trial.

The start of Scotland's National Naloxone Policy (NNP) in January 2011 [7], with funding for both NOR and community-based take-home naloxone (THN), had pre-empted the N-ALIVE trial's planned randomization in Scottish prisons. The primary outcome for Scotland's science-led NNP-evaluation [8] was a 20%–30% reduction in the proportion of opioid-related deaths (ORDs) with a 4-week antecedent of prison-release. As the proportion had been 10% in 2006–2010, Scotland's NNP had 80% power to discern reduction to 7% in 2011–13, as upper target; or to 8% in 2011–15, as lower target.

In this paper, we describe how external data for the third year of before/after evaluation of Scotland's NNP, a related public health policy [7], [8], [9], [10], were anticipated by eliciting prior opinion about the Scottish results in the month prior to their release as official statistics [11], [12]. We then describe how deliberations by N-ALIVE's Trial Steering-Data Monitoring Committee (TS-DMC) on N-ALIVE's own interim data, together with those on NOR from Scotland, led to the decision to cease randomization in the N-ALIVE pilot trial and to recommend to local Principal Investigators (PIs) that NOR be offered to already-randomized prisoners who had not yet been released [5].

To be ready to act promptly, we had elicited expert opinion about the Scotland's forthcoming results [11] in order to focus on the most probable scenarios for TS-DMC's decision-making. Unscheduled interim analysis was also undertaken of the N-ALIVE pilot trial's own data from returned prisoner self-questionnaires, specifically on the extent of NOR's administration intramuscularly to the ex-prisoner for whom it had been prescribed versus to another person.

We begin, therefore, with a brief history of formally eliciting prior opinion to inform the design and monitoring of RCTs funded by the UK's Medical Research Council (MRC); and some early accounts of DMC deliberations. The back-story on the NALoxone InVEstigation (N-ALIVE) follows, which puts our elicitation in context, and sets the scene for deliberations and decisions by the N-ALIVE's TS-DMC.

## 2 On elicitations for randomized trials funded by the Medical Research Council and deliberations by Data Monitoring Committees

The earliest example of formally eliciting prior opinion to inform trial design was “place your bets” about the mortality of surfactant-treated very premature babies (aged 25–29 weeks) in a RCT funded by MRC in the mid-1980s [13], [14]. This “trial roulette” method was again used in the design and early stopping of the MRC's neutron therapy trial in pelvic cancer [15], [16], [17], [18]: the minority prior belief on the relative mortality of neutrons versus photons turned out to have been consistent with trial's data. Following the early termination by the investigators of this neutron therapy trial, a decision later ratified by a specially-convened post-hoc DMC, the MRC required all of its RCTs to have a properly constituted DMC.

In 1994, Spiegelhalter, Freedman and Parmar [19] formalized Bayesian approaches to RCTs. Their Bayesian design and monitoring of the CHART trials included a description of how prior elicitation of clinicians' opinion could be used to form “enthusiastic” and “sceptical” prior distributions [20]. Neither CHART trial was closed to recruitment because, at each annual review, there was insufficient evidence to convert either the sceptics or the enthusiasts [21].

In 1999, on behalf of the Concorde, Alpha and Delta trials which randomized patients with asymptomatic HIV infection, Armitage (as DMC-chair) provided two insightful accounts of the DMC deliberations: the first on interpreting early data and trends in surrogate markers [22], [23] and the second as clear-cut differences in efficacy gradually emerged [24]. See also Wittes [25]; Ellenberg, Fleming and DeMets [26] for an early practical textbook; and the injunction by Grant that DMCs must show strong resolve when large unanticipated differences are inconsistent with existing evidence from outside the RCT [27], as Goodman later endorsed [28].

By 2005, the DAMOCLES Study Group, like the MRC, had recommended that every RCT should have a DMC [29]; and proposed a DMC charter to help them do their job well [30]. Of 20 questions that DAMOCLES posed to 25 regulatory or funding organizations, the two least likely to be answered were: on the training of DMC members (2 responses) and on decision-making within DMCs (3 responses) [29]. For further examples of DMC decision-making, see both the DAMOCLES Study Group [29] itself (four examples) and Pocock's editorial on when (not) to stop a clinical trial for benefit [31], in which he discussed the merit of the Haybittle-Peto boundary which requires P < 0.001 as evidence to stop an RCT for efficacy.

Tharmanathan et al. [32] surveyed the use of interim data (with or without the mention of DMCs) by RCTs published in eight major journals: of 1772 RCTs published during 2000–2005, 470 (27%) reported the use of a DMC and a further 116 (7%) some form of interim analysis without explicit mention of a DMC; see also Sydes et al. [33] (for the DAMOCLES Study Group) who had contrasted DMC-mentions in 1990 versus 2000.

## 3 Back-story on the N-ALIVE pilot trial and Scotland's National Naloxone Policy

In late summer 2008, the MRC funded the pilot phase (that is: first 10% of randomizations) of the N-ALIVE Trial [4], [5], [6] which was to run in two prison jurisdictions (Scotland; England & Wales). Pro-rata in each jurisdiction, 2800 consented eligible prisoners with a history of heroin-injection were to be randomized during incarceration to receive their assigned N-ALIVE pack on-release. The trial was double-blind only until participants opened their assigned N-ALIVE pack immediately after release.

The randomization ratio was 1:1. The N-ALIVE control packs contained no syringe and no naloxone. The naloxone packs contained a syringe of naloxone for “rescue” injection in the event that the participant overdosed on opioids [1], [2], [3]. The syringe contained 2 mg of naloxone hydrochloride in 2 ml of solution, for once-only intramuscular (IM) injection in the event of overdose. During information and consent sessions while incarcerated, all N-ALIVE participants were advised on how to administer 0.8 mg of naloxone.

The N-ALIVE pilot trial was designed to investigate the feasibility of randomized provision of NOR to eligible prisoners. The definitive N-ALIVE Trial would determine if NOR reduced participants' drug-related deaths (DRDs) by 30% in the first 4-weeks after release and by 20% in the subsequent 8 weeks [4], [5], [6]. Per 2800 releases in the control group, we expected 14 DRDs in the first 4-weeks and 3.5 DRDs in the subsequent 8 weeks [4], [5].

As high risk of overdose death soon after prison-release applies per-release, re-randomization was permitted provided that at least six months had elapsed since the participant's previous N-ALIVE release-date.

### 3.1 Contamination between randomized groups?

In designing the N-ALIVE pilot trial, we had anticipated contamination between randomized groups of up to 20% because participants who had been randomized to NOR might administer their naloxone alternatively to an opioid-dependent peer who had overdosed, some of whom - unknown to us - might have been randomized to N-ALIVE's control group.

Specifically, if ex-prisoners' 8 times higher DRD-risk in the first fortnight after release [1] was on account of an 8 times higher overdose-risk then, assuming that one of (say) three co-present injectors overdoses, there is an 80% chance that the person who overdosed was the recently-released ex-prisoner to whom naloxone (if also present) will therefore be administered.

However, if injectors' chance of opioid overdose is the same regardless of recent prison-release, so that recently-released ex-prisoners' DRD-risk is due to an 8 times higher fatality-rate per opioid overdose, then each of the injector-triad above has the same chance of opioid-overdose. In this scenario, there is potentially a two-thirds chance that the ex-prisoner's NOR is administered to another person so that contamination between N-ALIVE's randomized groups could be substantially higher than 20%: and especially so if, in addition, there is assortative mixing of recently-released prisoners.

Contamination was assessed in the N-ALIVE pilot trial by asking participants who were returned to prison to complete a returned prisoner self-questionnaire (RPSQ). The questionnaire asked participants about overdose soon after release, and whether naloxone was administered before the arrival of an ambulance; and also about their presence when someone else overdosed, and whether naloxone was administered before the arrival of an ambulance [5].

### 3.2 Pre-emption of N-ALIVE's randomization by National Naloxone Policies (NNPs) in Scotland and Wales

The decision in spring 2010 by Scotland's Minister for Safety and Communities to introduce Scotland's NNP from January 2011 pre-empted N-ALIVE's randomization in Scotland [7], [8].

Scotland became the first country in the world to have a funded public health policy of NOR for at-risk prisoners and THN for community-based opioid users. Wales [34], [35] followed Scotland's lead later in 2011, so that the N-ALIVE pilot trial could randomize in English prisons only. In May 2012 at Nottingham Prison, the N-ALIVE pilot trial randomized its first participants [6]. By the end of October 2014, 1570 participants had been randomized by 16 participating prisons in England.

### 3.3 Before/after evaluation of Scotland's National Naloxone Policy

Scottish ministers made provision for 33,000 naloxone-kits (at £11 per kit) to be issued during 2011–2013. In the event, the ministerial target took five years to be achieved and the cost per kit increased to £18 [9].

Based on Scotland's 1970 ORDs in 2006–2010 [36] versus around 1200 expected in 2011–2013 [8], the before/after evaluation of Scotland's NNP was designed to have 80% power to discern a 30% reduction in the proportion of prison-release ORDs (down from 10% to 7%) and a 20% reduction in the proportion of ORDs with a 4-week antecedent of prison-release and/or hospital-discharge (down from 20% to 16%).

Scotland's number of ORDs was not an effectiveness outcome for 2011–2013 because ORDs in Scotland (also in England and Wales [9]) had been on an age-related rising trajectory during the first decade of the 21st century [8], [12]. Moreover, ORDs were susceptible to sharp changes in the illicit heroin market.

## 4 Anticipatory elicitation

On behalf of Scotland's National Naloxone Advisory Group, the Information Services Division (ISD) in Scotland undertook the look-backs from Scotland's ORDs to establish their 4-week antecedent of i) prison-release and ii) prison-release and/or hospital-discharge. Findings were reported as official statistics [36]. Prior to 28 October 2014 [11], the baseline proportion of Scotland's ORDs in 2006–10 with a 4-week antecedent of prison-release and/or hospital-discharge had not been published; but was expected to be around 20%.

Table 1 shows the published information at the end of August 2014 [12], [36] on NNP's primary and secondary outcomes for the baseline period of 2006–2010; and for 2011–2013. With ministerial endorsement, Scotland had set regional targets for the community-issue of THN in 2013/14 so that Scotland's issued naloxone-kits in 2013 would almost surely exceed those in 2012 (and did [11]).

**Table 1:**
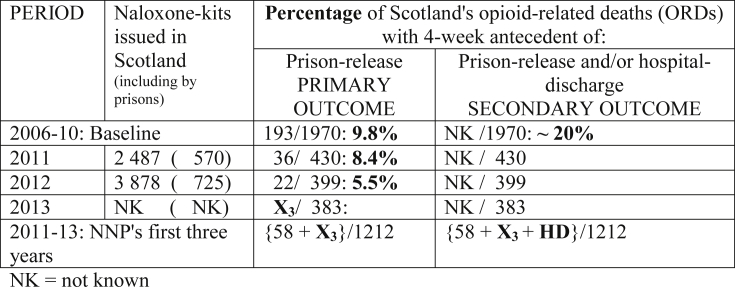
Published information by end August 2014 [9], [10] on the primary and secondary outcomes for Scotland's National Naloxone Policy (NNP): baseline period of 2006–2010 versus 2011–2013. **Before/after evaluation of Scotland's National Naloxone Policy & set-up for ELICITATION of unknown counts X**_**3**_**and HD where X**_**3**_ is the number of Scotland's 383 ORDs in 2013 with 4-week antecedent of prison-release and **HD** is the number of Scotland's 1212 ORDs in 2011–213 with 4-week antecedent of hospital-discharge but not prison-release.

Please see Supplementary Material for the invitation and elicitation-briefing that was issued to three cadres of individuals from whom we wished to elicit prior opinion: a) some 30 statistician-members of MRC Biostatistics Unit who attended a Workshop on Evidence Synthesis for Health in September 2014, b) four selected members of Scotland's National Naloxone Advisory Group and c) five members of the N-ALIVE pilot Trial team (its principal investigators; AMM; and trial-statistician). Even for biostatisticians, elicitation of expert opinion on a joint outcome was tricky as their low response-rate may indicate. The required joint outcome was: i) Scotland's number of ORDs in 2013 with a 4-week antecedent of prison-release (X_3_ in Table 2) and ii) Scotland's number of ORDs in 2011–2013 with a 4-week antecedent of hospital-discharge but **not** prison-release (HD in Table 2). Responses were received as follows: a) 11, b) 4 and c) 4. One of the 19 respondents offered an opinion only on i).

**Table 2:**
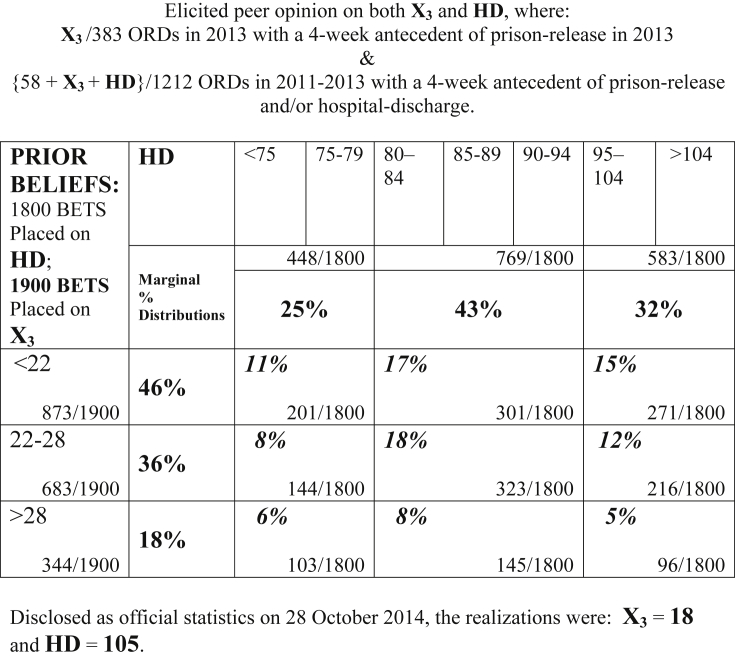
Summation of responses across three elicitation-sources.

Table 2 shows the summed responses from the above three elicitation-sources. The attention of the N-ALIVE pilot trial's TS-DMC was focused on the elicitation's three (out of nine) top-belief cells which together accounted for 50% of assessors' prior belief. As we learned subsequently on 28 October 2014, the actual outcomes were: i) 18 ORDs in 2013 with a 4-week antecedent of prison-release and ii) 181 ORDs in 2011–2013 with a 4-week antecedent of prison-release and/or hospital-discharge so that the number of ORDs in 2011–13 with a 4-week antecedent of hospital-discharge but **not** prison-release equalled 181-76, or 105 [9], [11].

## 5 Deliberations and decisions by N-ALIVE's Trial Steering-Data Monitoring Committee

In this section, we sketch the rationale for and main business of the N-ALIVE pilot trial's TS-DMC prior to October 2014. We then detail the TS-DMC's deliberations in October and November 2014.

The N-ALIVE pilot trial's emerging data on ORDs (now maximally for 2800 participants; versus 56,000 required to demonstrate NOR's a priori plausible effectiveness [4], [5], [8]) were highly unlikely to achieve a sufficient signal-noise ratio on effectiveness and so a joint TS-DMC had been appointed (which DA chaired). Membership included N-ALIVE's three co-principal investigators (JS, MKBP, SMB). The TS-DMC's independent chair and membership would, we hoped, form the nucleus of the main trial's DMC.

Prior to October 2014, the main business of the N-ALIVE pilot trial's TS-DMC had been to support the recruitment of a sufficient number of prisons in England; to deliberate on reasons for fewer prisoners with a history of heroin injection being identified by our prison-based N-ALIVE workers than anticipated by the number of prisoners engaged in drug treatment in 2005/06 [6]; and to engage governors in promoting their security staff's acceptance that our assigned N-ALIVE packs should be held with the prisoner's valuables [5], [6].

Our TS-DMC members had not expected to confront critical ethical decisions on whether to cease randomization in the N-ALIVE pilot trial. However, from the outset, their responsibilities did include the review of external evidence and recommending whether the pilot trial should continue to the N-ALIVE main trial.

The TS-DMC did not take formal action on NNP's interim data for 2011 + 2012 on prison-release ORDs, see Table 1, which had decreased significantly (p < 0.01) from 9.8% (193/1970) in 2006–2010 to 7.0% (58/829) in 2011 + 2012 as the wide confidence interval and NNP's before/after design warranted only a watching brief. Moreover, by the end of October 2013, the N-ALIVE pilot trial had received fewer than 70 of its own RPSQs.

In October 2014, however, TS-DMC members were faced by external data which required tense and careful deliberation. Prior elicitation of expert opinion had served to sensitize members to the sorts of decision that might have to be made. The elicitation results were considered by N-ALIVE's TS-DMC at its meeting on 20 October 2014, eight days ahead of ISD's official statistics release [11]. The TS-DMC was also made aware of commissioners' plans for prisons in the North West region of England to issue NOR to eligible prisoners; and that, if funding for the N-ALIVE pilot trial was not to be extended beyond March 2015, randomizations would have to cease by the end of December 2014 to allow time for 12-weeks’ follow-up and an orderly closure of the N-ALIVE sites.

The TS-DMC minutes recorded that, following the release of the third year of results on Scotland's NNP [11] on 28 October 2014, a teleconference would be organized to decide the course of action for N-ALIVE. In practice, the TS-DMC chair asked that the TS-DMC's initial deliberations be conducted by email as an analytical summary of Scotland's 3-year results for 2011–2013 had to be prepared. Also, in view of the late registration of coroner-referred deaths in England and Wales, information on ORDs in Wales and England by death-year, rather than by the year of death-registration, were needed to set Scotland's ORDs in context [9], [37], [38].

As Scotland's 3-year results coincided with one of the three cells in Table 2 wherein lay 50% of the prior belief, the elicitation exercise had served mainly as reassurance that prior beliefs and realization were not at odds. There was no added scepticism [19], [20], [21] to be weighed in the balance.

### 5.1 Deliberations: 28 October to 18 November 2014

Key considerations were:

a) N-ALIVE pilot trial's undertaking to participants and to the Research Ethics Committee that they would be notified immediately if the principal investigators had reason to believe that the N-ALIVE main trial could not go ahead as planned;
b) N-ALIVE pilot trial's own data from RPSQs, together with Scotland's information from re-supplies, showed that NOR was administered to **another: self** in the ratio **15:5** and **21:12** (that is: 36:17 or 2:1), giving an upper 99% confidence limit of 50% for NOR's administration to the recently-released prisoner as assigned;
c) Effect-size for Scotland's primary outcome of prison-release ORDs (p < 0.001) [31] was consistent with the a priori targets adopted by the N-ALIVE main trial but Scotland's recently-released prisoners potentially benefitted from community-issued THN as well as from NOR;
d) Association is not causation, and so Scotland's before/after comparison needed to be appraised in the light of Hill's criteria on causality [9], [39];
e) Scotland's NNP outcomes were published annually as official statistics but a formally peer-reviewed report on NNP's outcomes in 2011–13 versus 2006–10 [9] was lacking;
f) As Europe's largest prison-based randomized controlled trial (RCT) [6], if the N-ALIVE pilot trial ceased randomizing, damage could be done internationally to the case for prison-based RCTs;
g) As the N-ALIVE trial's participants were held in prison custody, the highest ethical standards would be maintained by TS-DMC on their behalf;
h) The likely consequence in England - if the N-ALIVE pilot trial ceased randomizing eligible prisoners in the ratio 1:1 between NOR and control - was that no eligible prisoner in England would receive NOR rather than, as now, half of those who participated from N-ALIVE prisons;
i) Hence, continued prison-based randomization would be ethical on the basis that NOR was a rationed or restricted resource;
j) Upon release, N-ALIVE's participants are as free as any other citizen is to obtain THN by prescription, for example by requesting it from their general practitioner;
k) Finally, N-ALIVE's principal investigators were scientifically-bound to consider and promote randomized alternatives, such as randomized step-wedge designs, to ensure that as robust evidence as possible could be got [40], [41] from the instigation of regional NNPs in England.

Considerations c) to e) were addressed by SMB's drafting for consideration by TS-DMC of a 3-year report on Scotland's NNP outcomes. This report, which included the application of Hill's criteria on causality [39], formed the basis of a co-authored, peer-reviewed subsequent publication in *Addiction* [9]. Considerations f) to k) were addressed by JS's drafting of an ethical and wider scientific counter-case for continued randomization.

Considerations f) and g) were balanced by considerations i) and j). The fairness of continued randomization when a resource such as NOR is scarce or restricted is a strong argument, both scientifically and ethically. TS-DMC's duty is, however, primarily to participants in the trial which the TS-DMC was convened to oversee [29].

Considerations h) and k) were met by the N-ALIVE co-principal investigators' letter to England's Chief Medical Officer to appraise her of the Scottish data, the decisions to be taken in respect of the N-ALIVE pilot trial and the likely cost-effectiveness of a NNP in England which should aim to issue 9000 to 20,000 naloxone-kits per annum [8], including NOR for eligible prisoners as in Scotland [9], [42] and Wales [35].

### 5.2 Decisions: 18 November to 1 December 2014

According to its charter [6], the N-ALIVE pilot trial's TS-DMC was quorate at its meeting on 18 November 2014 because two independent members (DA and SW) were present in addition to the three principal investigators. But, because the TS-DMC was considering a major action, the TS-DMC chair needed to communicate with the absent members (JP and JRR) as soon after the meeting as possible to check if they were in agreement. If not, a further teleconference should be arranged with the full TS-DMC.

Considerations a) and b) were deciding factors: we estimated that at least half the administrations of NOR in the N-ALIVE main trial would be to some-one other than the ex-prisoner for whom NOR had been prescribed. Even without NOR-administered contamination of the N-ALIVE control group, the number to be randomized in the N-ALIVE main trial would be excessive (over 150,000). Worse, substantial contamination, in excess of 20%, could not be ruled out and so the main trial could not go ahead.

The N-ALIVE pilot trial's TS-DMC decided, on the basis of a) and b), that randomization should cease in the N-ALIVE pilot trial on 8 December 2014. Because of the strength of the evidence from Scotland's NNP (since peer-reviewed [9]) and the WHO Guidelines published on 5th November 2014 [40], [41], the TS-DMC recommended to local PIs that, once randomization ceased, all randomized participants who remained in prison should be offered NOR (ie those who had been allocated to control as well as those assigned to NOR). Submission to the Research Ethics Committee was made on 21 November 2014, which notified its approval on 1 December 2014 of the TS-DMC's recommendations and N-ALIVE's updated information for participants on the basis for its decisions (see http://www.ctu.mrc.ac.uk/our_research/research_areas/other_conditions/studies/n_alive/). The TS-DMC's recommendations were also endorsed by N-ALIVE's prison-based investigators who put them into force.

## 6 Concluding remarks

The elicitation of expert opinion on the Scottish NNP's likely outcomes during 2011–13, when they would be available anyway in less than two weeks, set the scene for the TS-DMC's deliberations but was not otherwise influential, because prior beliefs and realization were consistent; not contradictory.

Time for reflection and appraisal of causality were necessary for TS-DMC's decisions to have been reached unanimously on what to do in respect of serving prisoners who had been randomized to the control group.

The critical decision – that an individually-randomized controlled trial of naloxone-on-release had been shown to be the wrong design because at most half the administrations of NOR were to the ex-prisoner as assigned – was more easily made. The elicitation of expert opinion had no bearing on this decision, for which the dominant consideration was consistency of **another: self** ratio between N-ALIVE's RPSQs and Scotland's information from NOR re-supplies.

## Declaration of interests

**DA, AMM, JP, MKBP:** no conflicts of interest.

**JS:** JS is a researcher and clinician who has worked with a range of types of treatment and rehabilitation service-providers, including treatments within prison and on prison release. JS is supported by the National Institute for Health Research (NIHR) Biomedical Research Centre for Mental Health at South London and Maudsley NHS Foundation Trust and King's College London. He has also worked with a range of governmental and non-governmental organizations, and with pharmaceutical companies to seek to identify new or improved treatments (including naloxone products) from whom he and his employer (King's College London) have received honoraria, travel costs and/or consultancy payments. This includes work with, during past 3 years, Martindale, Reckitt-Benckiser/Indivior, MundiPharma, Braeburn/MedPace and trial medication supply from iGen. His employer (King's College London) has registered intellectual property on a novel buccal naloxone formulation with which JS is involved. JS has also been named in a patent registration by a Pharma company as inventor of a concentrated nasal naloxone spray. For a fuller account, see JS's web-page at http://www.kcl.ac.uk/ioppn/depts/addictions/people/hod.aspx.

**SMB:** SMB served on Scotland's National Naloxone Advisory Group and co-authored the peer-review paper on before/after evaluation at 3-years of Scotland's National Naloxone Policy. SMB holds GlaxoSmithKline shares.

**JRR:** JRR chaired Scotland's National Forum on Drugs-Related Deaths which recommended that Scotland should have a National Naloxone Policy.

**SW:** SW is co-author of British Association for Psychopharmacology evidence-based guidelines for the management of substance abuse, harmful use, addiction and co-morbidity.

## Funding and role of funding source

The pilot N-ALIVE Trial was grant-funded by the Medical Research Council (MC_G0800012) and co-ordinated by the MRC Clinical Trials Unit at University College London, which core-funds MKBP and AMM.

SMB was funded by Medical Research Council programme number MC_U105260794.

JS is core-funded by his university, King's College London.

The MRC's funding board had no role in data analysis, data interpretation, the decision to cease randomization in the N-ALIVE pilot Trial, the decision to submit for publication this account of how the decision was reached, the writing of this report.

The corresponding author had full access to all the data in the study and had final responsibility for the decision to submit for publication.

## Authors' contributions

Elicitation concept and design: SMB.

Implications of prisoners' altruism for feasibility of individually-randomized N-ALIVE main trial: MKBP.

Liaison with Office for National Statistics to obtain information on opioid-related deaths for England and Wales by calendar year of death, as part of applying Hill's criteria for determining causation in Scotland's NNP: SMB.

Case for continued randomization in the wider public interest: JS.

Deliberations by independent TS-DMC members: DA (chair), JP, JRR, SW.

Oversight of MRC-CTU's minutes on TS-DMC decisions and giving effect to them by liaison with Research Ethics Committee and with principal investigators at N-ALIVE prisons: AMM.

Initial drafting of paper: SMB.

Editing, interpretation and discussion: all authors.
