# Supplementary material for: External data required timely response by the Trial Steering-Data Monitoring Committee for the NALoxone InVEstigation (N-ALIVE) pilot trial
Source: Contemp Clin Trials Commun. 2017 Jan 18;5:100–6. doi: 10.1016/j.conctc.2017.01.006 (PMC5389338; doi:10.1016/j.conctc.2017.01.006)
Supplement: Supplementary file 1 [file mmc1.docx]

**Supplementary Material**

**Elicitation of prior opinion about Scotland’s 3-year results on 28 October 2014 from its National Naloxone Policy.**

1. Attached is  a briefing in which the results for Scotland's primary outcome for 2011+2012 are summarized where:
2. **Primary**  outcome: % opiate-related deaths (ORDs) with a 4-week antecedent of **prison-release**; primary outcome in the 5-year baseline period was **10%** (193 of 1970 ORDs).

*Secondary* outcome: % opiate-related deaths (ORDs) with a 4-week antecedent of **prison-release** or *hospital-discharge*.

PLEASE NOTE: The numerator for secondary outcome in 2006-2010 has not been disclosed but is believed to be around *20%* of Scotland's 1970 ORDs in 2006-2010.

1. We can expect that the effectiveness of NNP should accelerate from 2011 thro’ 2012 thro’ 2013 because some of the naloxone-kits issued in 2011 will still be available for use in subsequent years; similarly for 2012-issued naloxone-kits; and for the 2013-issue . . .

PLEASE NOTE: More or less sophisticated simulation studies can be run to estimate the extent of this acceleration, one version of which is 1:3:4 from 2011 thro’ 2013.

1. Although a similar number of naloxone-kits was issued in each of the policy’s first two financial years 2011/2012 and 2012/2013 (about 3,600 each), the roll-out in the calendar year of 2011 had a slow start, with very limited issue in the first quarter of 2011.
2. Many more naloxone-kits should have been issued in 2013/14 (approximately 5,000) because targets were set for Scotland's community-based issue of THN-kits.
3. Target for prisons' issue of NOR-kits did not apply until 2014/15.
4. Unlike prisons, Scotland's hospitals are not specifically tasked to issue THN-kits to opiate-dependent patients whom they discharge: despite their 4-week ORD-risk being high, albeit only half the risk for similar clients in the 4-weeks after prison-release.

**PLEASE PLACE YOUR BETS!**

With the above information in mind, please place **100 bets** in the 21-cell grid below for the joint-outcomes of **X_3_** & **HD**, where

**X_3_** = primary outcome's **numerator** in 2013 when the denominator was 383 ORDs

& where

58 + **X_3_** + **HD**= numerator for *secondary* outcome for 2011+2012+2013, for which the KNOWN denominator is 430+399+383 = 1212 ORDs for the calendar years of 2011+2012+2013.

| PRIOR BELIEF: 100 BETS | **HD**  < 75 | 75 – 79 | 80 - 84 | 85- 89 | 90- 94 | 95– 104 | **HD** >104 |
| --- | --- | --- | --- | --- | --- | --- | --- |
| **X_3_** < 22 |  |  |  |  |  |  |  |
| 22 -28 |  |  |  |  |  |  |  |
| > 28 |  |  |  |  |  |  |  |

Please also complete:

Age-group of respondent: **1** = 20-29; **2** = 30-39; **3** = 40-49; **4** = 50+ years [ ]
